# Determinants of patient preferences for total knee replacement: African-Americans and whites

**DOI:** 10.1186/s13075-015-0864-2

**Published:** 2015-12-03

**Authors:** C. Kent Kwoh, Ernest R. Vina, Yona K. Cloonan, Michael J. Hannon, Robert M. Boudreau, Said A. Ibrahim

**Affiliations:** University of Arizona School of Medicine and University of Arizona Arthritis Center, 1501 N. Campbell Ave., PO Box 245093, Tucson, Arizona 85724 USA; University of Pittsburgh School of Public Health, 130 De Soto St., 127 Parran Hall, Pittsburgh, Pennsylvania 15261 USA; University of Pittsburgh School of Medicine, 130 North Bellefield Ave., 4th Floor, Pittsburgh, Pennsylvania 15213 USA; University of Pennsylvania Perelman School of Medicine and Philadelphia VA Medical Center, 3900 Woodland Ave., Philadelphia, Pennsylvania 19104 USA

**Keywords:** Osteoarthritis, Knee replacement surgery, Race, Health disparity, Treatment preference

## Abstract

**Introduction:**

Patient preferences contribute to marked racial disparities in the utilization of total knee replacement (TKR). The objectives of this study were to identify the determinants of knee osteoarthritis (OA) patients’ preferences regarding TKR by race and to identify the variables that may mediate racial differences in willingness to undergo TKR.

**Methods:**

Five hundred fourteen White (WH) and 285 African-American (AA) patients with chronic knee pain and radiographic evidence of OA participated in the study. Participants were recruited from the community, an academic medical center, and a Veterans Affairs hospital. Structured interviews were conducted to collect socio-demographics, disease severity, socio-cultural determinants, and treatment preferences. Logistic regression was performed, stratified by race, to identify determinants of preferences. Clinical and socio-cultural factors were entered simultaneously into the models. Stepwise selection identified factors for inclusion in the final models (*p* < 0.20).

**Results:**

Compared to WHs, AAs were less willing to undergo TKR (80 % vs. 62 %, respectively). Better expectations regarding TKR surgery outcomes determined willingness to undergo surgery in both AAs (odds ratio (OR) 2.08, 95 % confidence interval (CI) 0.91-4.79 for 4^th^ vs. 1^st^ quartile) and WHs (OR 5.11, 95 % CI 2.31-11.30 for 4^th^ vs. 1^st^ quartile). Among AAs, better understanding of the procedure (OR 1.80, 95 % CI 0.97-3.35), perceiving a short hospital course (OR 0.81, 95 % CI 0.58-1.13), and believing in less post-surgical pain (OR 0.73, 95 % CI 0.39-1.35) and walking difficulties (OR 0.66, 95 % CI 0.37-1.16) also determined willingness. Among WHs, having surgical discussion with a physician (OR 1.96, 95 % CI 1.05-3.68), not ever receiving surgical referral (OR 0.56, 95 % CI 0.32-0.99), and higher trust in the healthcare system (OR 1.58, 95 % CI 0.75-3.31 for 4^th^ vs. 1^st^ quartile) additionally determined willingness. Among the variables considered, only knowledge-related matters pertaining to TKR attenuated the racial difference in knee OA patients’ treatment preference.

**Conclusions:**

Expectations of surgical outcomes influence preference for TKR in all patients, but clinical and socio-cultural factors exist that shape marked racial differences in preferences for TKR. Interventions to reduce or eliminate racial disparities in the utilization of TKR should consider and target these factors.

**Electronic supplementary material:**

The online version of this article (doi:10.1186/s13075-015-0864-2) contains supplementary material, which is available to authorized users.

## Introduction

Osteoarthritis (OA) is a highly prevalent and disabling disease, and the lifetime risk of developing knee OA is one in two [1–3]. When conservative therapies no longer provide lasting pain relief, total knee replacement (TKR) becomes a very effective treatment option [4]. Despite its efficacy, there are marked racial disparities in the utilization of TKR. Using Medicare claims data, it has been estimated that the annual rate of TKR was 4.84 per 1000 among African-American (AA) women, compared to 5.97 per 1000 among white (WH) women [5]. The rate for AA men (1.84 per 1000) was also lower than that for WH men (4.82 per 1000). Analyzing Medicare data from other time periods and data from other national databases have elicited similar findings [5–8].

Reasons for racial disparities in TKR utilization rate are complex and involve patient-level, provider-level, and system-level factors [7]. Patient preference has emerged as a key factor, however. It is a strong predictor of time to receipt of a first total joint replacement [9]. Patient preference in joint replacement may also vary by race, sex, and other sociodemographic factors [10–16]. In studies of veterans with advanced knee or hip OA, AAs were consistently less willing to consider joint replacement compared to WHs [10–13]. In a focus group study of AA patients’ attitudes and preferences in arthritis care, AAs expressed preferences for natural remedies and against undergoing surgery [17].

Patient preference is largely an attitudinal disposition [7]. According to the expectancy-value model, attitudes arise spontaneously and inevitably as we form beliefs about an object [18]. Our overall attitude towards an object is determined by the subjective value of the object’s attributes in relation to the depth of the association [18]. Hence, racial differences in treatment preferences in TKR are likely due to varying beliefs or opinions about the procedure and the healthcare system. Studies have shown racial variations in knowledge and expectations of joint replacement [11, 19–21]. WHs are more likely to believe that TKR is efficacious and are more likely to be familiar with the procedure than AAs [11, 20, 21]. AAs are more likely to expect surgical complications [11, 21]. AAs are also less likely to be satisfied with their communication with orthopedic surgeons than WHs [22].

Moreover, studies have investigated some of the correlates of willingness to undergo joint replacement. Patients’ perceptions of appropriateness of surgery and of high efficacy and acceptable risks when undergoing joint replacement are all strongly associated with willingness to consider the procedure [16, 20]. Discussing the procedure and receiving a recommendation from a physician may also determine willingness to undergo joint replacement [15, 20]. High trust in physicians may also increase willingness [20]. Despite such evidence, the specific socio-cultural beliefs that impact patients’ willingness to consider TKR as a treatment option, by patient race, remain unclear, as do the factors that explain racial differences in treatment preferences in TKR.

The primary objective of this study is to examine the roles of patients’ knowledge of TKR, beliefs about TKR, attitudes towards TKR, attitudes towards providers, and attitudes towards the healthcare system, in determining willingness to undergo TKR, by patient race. The secondary objective is to determine which of these factors could mediate racial differences in willingness to undergo TKR.

## Methods

Participants were recruited from the University of Pittsburgh and the Veterans Affairs (VA) Pittsburgh Healthcare System clinics. They were also sought through mailings and local advertisements. The recruitment methodology has been previously reported [23]. The study was approved by the University of Pittsburgh and VA Institutional Review Boards. Informed consent was obtained from all participants in the study.

Inclusion criteria included: AA or WH race, age ≥50 years, presence of chronic frequent knee pain [24], moderate-to-severe knee OA based on Western Ontario McMaster Index (WOMAC) summary score ≥39 [25], radiographic evidence of OA (i.e., Kellgren-Lawrence grade ≥2) [26], and presence of knee OA according to American College of Rheumatology [27] criteria. Exclusion criteria included: history of major joint replacement, terminal illness, inflammatory arthritis, or dementia. Patients participated in a face-to-face interview.

### Key study variables

To determine willingness to undergo TKR, participants were asked: “If your knee pain were ever to get severe, would you be willing to have surgery to replace your knee if your doctor recommended it?” [28]. This item used a five-category ordinal response scale. Responses were dichotomized to willing (“definitely willing” and “probably willing”) or unwilling (“unsure,” “probably not willing” and “definitely not willing”) [12, 23]. The primary predictor variable was self-identified race (AA or WH).

### Study covariates

#### Socio-demographics

Socio-demographic characteristics included age, sex, marital status, household income, education, employment status, and type(s) of health insurance. Functional social support was measured using the 5-item modified Medical Outcomes Study-social support scale (MOS-SSS) [29]. Higher scores suggest more social support (range 0–20).

#### Clinical information

OA-related disease severity was assessed using the 24-item WOMAC [25]. Higher summary scores indicate increased pain, stiffness and functional limitations (range 0–100). Comorbid conditions were weighted and summed using a modified Charlson co-morbidity index [30]. Quality of life was assessed using the Short Form Health Survey (SF-12v2, range 0–100) [31]. Depression was assessed using the Patient Health Questionnaire-9 (PHQ-9, range 0–27) [32].

### Potential mediator variables

#### Religiosity

Religiosity was measured using a 4-item scale that assesses people’s religious behavior and self-identification [33]. Higher scores indicate greater levels of religiosity (range 0–14).

#### Knowledge of TKR

To assess familiarity with TKR, and perceptions of benefits and risks of TKR, participants were asked questions that were previously used to assess knowledge (3 items) and perceptions (5 items) of joint replacement [11]. To assess TKR outcome expectations, we used the Hospital for Special Surgery (HSS) Knee Replacement Expectations Survey [34]. The total score ranges from 0–76, with 76 being the highest expectation.

#### TKR utilization process

The following yes/no questions were asked: 1) “Has your doctor ever discussed surgery to replace your knee?”; 2) “Has your doctor ever referred you to an arthritis specialist?”; 3) “Has your doctor ever referred you to a surgeon that specializes in arthritis?”; 4) “Have any of your doctors ever recommended surgery to replace your knee?”

#### Trust in providers and healthcare system

Trust in physicians was determined using an 11-item scale (range 11–55) [35]. Trust in the healthcare system was assessed using the 9-item Health Care System Distrust measure (range 9–45). Higher summary scores indicate stronger trust in both measures.

### Statistical analysis

Socio-demographic and clinical characteristics were compared by race, using *t* tests or Wilcoxon rank-sum tests for continuous variables and Pearson chi square (χ^2^) tests for categorical variables. In the same manner, all patient-reported knowledge, attitudes and beliefs measured were contrasted by willingness.

#### Association of race with willingness to undergo TKR

Unadjusted and adjusted logistic regression models were used to evaluate the association of willingness and race. Covariates for adjusted models were selected *a priori*, which included recruitment site, age, sex, income, and WOMAC total score [12]. Additional covariates considered for adjusted models included education, employment, health insurance, comorbidities, social support, and mental health status. Variables associated with both willingness and race (*p* <0.10) in bivariate comparisons were entered simultaneously into the model. Forward stepwise selection (*p* <0.10) was used to select covariates to include in a final adjusted model. Statistical significance for the main effect of race on willingness was calculated using likelihood ratio (LR) tests.

#### Association of potential mediators with willingness to undergo TKR

Separate adjusted logistic regression models were used to assess the association between willingness to undergo TKR and religiosity, trust in physicians, trust in healthcare, expectations of TKR, familiarity with TKR, perceptions of benefits and risks of TKR, and TKR utilization process measures. Because a summary score for the TKR-related knowledge measures (except for expectations of TKR) were unavailable, all TKR-related knowledge items were entered simultaneously into a multivariable model. Stepwise selection (*p* <0.20) was used to identify knowledge-related items and TKR utilization process items to be retained in subsequent models. Statistical significance was evaluated using LR tests.

Next, potential mediators were entered simultaneously into a single model. Only TKR knowledge items and utilization process items that were significantly associated with willingness to undergo TKR (based on previous models) were considered for inclusion. Stepwise selection (*p* <0.20) was used to identify which variables to include in subsequent models. An LR test was used to test whether these variables were associated with willingness. Analyses were run using data from all participants then stratified by race. Any variable associated with willingness to undergo TKR in more than one model (full, WHs only, AAs only) was selected for inclusion in the mediation model.

#### Mediation analysis

Potential mediators were entered (individually and simultaneously) into the initial adjusted logistic regression model (i.e., willingness = race + covariates + mediator(s)). Any item identified by at least one of the stepwise models described above was included as a mediating variable in the mediation models. Attenuation of the estimated odds ratios before and after inclusion of the variables (>10 % change) provided evidence for mediation of the association between race and willingness to undergo TKR [36]. In all regression models, 95 % confidence intervals were calculated using robust variance estimates. Statistical significance was set with α of 0.05. Analyses were performed using STATA 12.0 (StataCorp LP).

## Results

Participants included 514 WH and 285 AA patients with knee OA. AA participants were younger and more likely to be unemployed or disabled (Table 1). At screening, they also had higher WOMAC total, higher PHQ-9 depression, and lower SF-12 physical health scores.

**Table 1:** Participant characteristics by race

Characteristic	White (n = 514)	African-American (n = 285)	*P* value^a^
Socio-demographic characteristics			
Age, mean ± SD, years	64.54 ± 9.39	58.68 ± 8.13	<0.001
Sex, n (%) female	302 (58.8)	207 (72.6)	<0.001
Currently married, n (%)	275 (53.5)	65 (22.8)	<0.001
Education, n (%)			<0.001
≤High school or GED	200 (39.1)	170 (60.1)	
Post-secondary training, Associates/Bachelors degree	194 (38.0)	98 (34.6)	
Graduate degree	117 (22.9)	15 (5.3)	
Income, n (%)			<0.001
<$10,000	32 (6.9)	84 (31.7)	
$10,000-$19,999	72 (15.6)	83 (31.3)	
$20,000-$29,999	75 (16.2)	35 (13.2)	
$30,000-$39,999	50 (10.8)	17 (6.4)	
$40,000-$49,999	51 (11.0)	12 (4.5)	
≥$50,000	183 (39.5)	34 (12.8)	
Employment status, n (%)			<0.001
Full time	145 (28.4)	70 (24.6)	
Part time	51 (10.0)	23 (8.1)	
Unemployed	36 (7.0)	46 (16.2)	
Disabled	53 (10.4)	81 (28.5)	
Retired	226 (44.2)	64 (22.5)	
Health insurance^b^, n (%)			
Medicare	225 (46.2)	98 (35.5)	0.004
Medicaid	53 (11.2)	63 (22.9)	<0.001
HMO or private/group	381 (77.6)	161 (57.7)	<0.001
No insurance	12 (2.6)	21 (7.7)	0.001
MOS social support, mean ± SD	15.17 ± 4.79	13.44 ± 5.26	<0.001
Recruitment site, n (%) non-Veterans Affairs	467 (90.9)	278 (97.5)	<0.001
Clinical characteristics			
WOMAC , mean ± SD	42.98 ± 15.27	53.98 ± 15.10	<0.001
Comorbidities, mean number ± SD	3.22 ± 1.71	3.56 ± 1.69	0.007
PHQ-9 depression, mean ± SD	4.77 ± 4.67	6.63 ± 5.5	<0.001
SF-12 physical health, mean ± SD	38.50 ± 11.01	36.25 ± 10.00	0.0045

^a^ *P* value from Pearson chi square or *t* test. ^b^Individuals may be included in multiple categories. *MOS* Medical Outcomes Study, *PHQ-9* Patient Health Questionnaire, *SF* MOS 12-Item Short Form Health Survey, *WOMAC* Western Ontario McMaster Index, *GED* General Educational Development test, *HMO* Health Maintenance Organization

### Bivariate associations between potential mediations and willingness

Among all participants, several TKR-related knowledge items were associated with willingness to undergo surgery, including having family/friend who had had joint surgery and reporting a good understanding of TKR (Table 2). Believing in a shorter duration of hospital stay (*p* = 0.023), lower post-surgical pain (*p* <0.001), and less difficulty walking after surgery (*p* <0.001) were all associated with willingness to consider TKR. Those willing to undergo TKR also had a higher score for expectations of HSS knee replacement (*p* <0.001) and higher scores for trust in physicians (*p* = 0.034) compared to those who were unwilling to undergo TKR.

**Table 2:** Characteristics of OA patients by willingness to undergo TKR

	Not willing (n = 208)	Willing (n = 585)	*P* value^a^
Socio-demographic Characteristics			
Race, n (%) African-American	106 (51.0)	176 (30.1)	<0.001
Age, mean ± SD, years	62.88 ± 10.64	62.28 ± 8.87	0.467
Education, n (%)			0.008
≤High School or GED	115 (55.3)	254 (43.4)	
Post-secondary/Associates/Bachelors	69 (33.2)	219 (37.4)	
graduate degree	22 (10.6)	109 (18.6)	
Income, n (%)			<0.001
<$10,000	39 (21.3)	76 (14.1)	
$10,000-$19,999	56 (30.6)	97 (18.0)	
$20,000-$29,999	21 (11.5)	88 (16.3)	
$30,000-$39,999	19 (10.4)	48 (8.9)	
$40,000-$49,999	16 (8.7)	47 (8.7)	
≥$50,000	32 (17.5)	183 (34)	
Employment status, n (%)			0.006
Full time	40 (19.2)	174 (29.9)	
Part time	17 (8.2)	57 (9.8)	
Unemployed	34 (16.3)	48 (8.3)	
Disabled	40 (19.2)	92 (15.8)	
Retired	77 (37.0)	210 (36.1)	
Health insurance^b^, n (%)			
Medicare	93 (46.3)	227 (40.8)	0.181
Medicaid	37 (19.0)	78 (14.2)	0.114
Private/group	81 (42.0)	292 (52.9)	0.009
No insurance	14 (7.6)	19 (3.5)	0.021
Recruitment site, n (%) non-Veterans Affairs	196 (94.2)	543 (92.8)	0.2247
Knowledge, attitudes and beliefs			
Religiosity, mean ± SD	10.19 ± 3.78	9.59 ± 4.00	0.053
Knowledge of TKR: familiarity, n (%) yes			
Heard about hip/knee surgery	158 (76.7)	458 (79.0)	0.497
Family or friend who had hip/knee surgery	160 (76.9)	498 (85.1)	0.007
Good understanding of knee replacement	106 (51.0)	365 (64.9)	0.001
Knowledge of TKR: risks and benefits			
How often death from knee replacement, n (%)			0.257
Never	29 (14.7)	75 (13.4)	
Extremely rare	134 (67.7)	406 (72.2)	
Sometimes	32 (16.2)	79 (14.1)	
Often	3 (1.5)	2 (0.4)	
How long in hospital after knee replacement, n (%)			0.023
1 to 3 days	57 (28.2)	199 (34.6)	
4 to 7 days	79 (39.1)	250 (43.5)	
1 to 2 weeks	45 (22.3)	84 (14.6)	
>2 weeks	21 (10.4)	42 (7.3)	
How long to recover from knee replacement, n (%)			0.378
<2 weeks	1 (0.5)	3 (0.5)	
2 weeks to 1 month	9 (4.5)	30 (5.2)	
1 to 2 months	44 (22.1)	140 (24.3)	
2 to 6 months	78 (39.2)	233 (40.4)	
6 to 12 months	50 (25.1)	146 (25.3)	
>12 months	17 (8.5)	25 (4.3)	
How much pain after recovery, n (%)			<0.001
None	17 (8.5)	87 (15.4)	
A little	74 (37.0)	267 (47.2)	
A moderate amount	98 (49.0)	205 (36.2)	
An extreme amount	11 (5.5)	7 (1.2)	
How much difficulty walking after recovery, n (%)			<0.001
None	14 (6.9)	120 (21.0)	
A little	98 (48.0)	306 (53.5)	
A moderate amount	84 (41.2)	134 (23.4)	
An extreme amount	8 (3.9)	12 (2.1)	
Expectations of TKR, mean ± SD	44.48 ± 17.26	53.40 ± 13.90	<0.001
TKR utilization process, n (%) yes			
Doctor ever discussed surgery	65 (31.6)	213 (36.5)	0.204
Referred to arthritis specialist	67 (33.0)	172 (29.9)	0.404
Referred to surgeon	101 (49.3)	268 (47.0)	0.580
Doctor recommended surgery	47 (22.8)	130 (22.4)	0.906
Trust in physicians, mean ± SD	38.62 ± 6.75	39.77 ± 6.65	0.034
Trust in healthcare system, mean ± SD	26.11 ± 6.17	25.32 ± 5.55	0.090

^a^ *P* value from Pearson chi square or *t* test. ^b^Individuals may be included in multiple categories. *TKR* total knee replacement, *GED* General Educational Development test

In stratified analyses, expectations of having ‘none’ or ‘a little’ difficulty walking after surgical recovery were related to willingness to undergo TKR among AA and WH participants (Additional files 1 and 2). Expectations of having ‘none’ or ‘a little’ pain after TKR was associated with willingness only among AAs (Additional file 1). Having a discussion about surgery with a doctor was associated with willingness only among WHs (Additional file 2). Higher scores for expectations of HSS knee replacement were associated with willingness to undergo TKR in both AA (*p* <0.001) and WH participants (*p* <0.001).

Willingness to undergo TKR was less often endorsed by AAs (62 %) as compared with WHs (80 %), even after adjustment for recruitment site, sex, age, income, WOMAC total score, presence of private/group health insurance, and social support (adjusted odds ratio (OR) 0.43, 95 % CI 0.28, 0.67). We also found a significant interaction between race and sex but the interaction was no longer significant in a fully adjusted model (Additional file 3).

### Multivariate models: associations of potential mediators with willingness

After adjustment for recruitment site, sex, age, income and WOMAC total score, higher expectations of knee replacement was associated with willingness in the overall sample to undergo TKR and when stratified by race (Table 3). Among all participants, greater trust in physicians was also significantly associated with willingness even when adjusted for the same covariates (*p* = 0.047). Stepwise logistic regression in the full study sample showed that the following TKR-related knowledge items were associated with willingness to undergo TKR, after adjustment for the covariates: having family/friend who had knee/hip surgery, having a good understanding of TKR, perceiving less pain after surgery, and perceiving less difficulty walking after surgery (*p* <0.0001). These individual items and perceived decreased length of hospital stay were selected for inclusion in the mediation models (Additional file 4). Using the same process, the following TKR utilization process items were also selected for inclusion in the mediation models: having a discussion about surgery with a doctor, having been referred to an arthritis specialist, and not having been referred to a surgeon (Additional file 4).

**Table 3:** Association by race between willingness to undergo TKR and religiosity, expectations of knee replacement, trust in physicians, and trust in healthcare

	All		White		African-American	
	OR (95 % CI)^a^	Wald Test^b^	OR (95 % CI)^a^	Wald Test^b^	OR (95 % CI)^a^	Wald Test^b^
Religiosity^c^	0.91 (0.77, 1.07)	0.2441	1.09 (0.89, 1.35)	0.404	0.79 (0.60, 1.06)	0.114
Expectations regarding Total knee replacement^c^	1.54 (1.31, 1.82)	<0.001	1.63 (1.28, 2.07)	<0.001	1.48 (1.16, 1.90)	0.002
Trust in physicians^c^	1.17 (1.00, 1.36)	0.047	1.17 (0.94, 1.44)	0.155	1.23 (0.98, 1.55)	0.078
Trust in healthcare^c^	0.88 (0.75, 1.04)	0.130	1.03 (0.82, 1.31)	0.778	0.80 (0.62, 1.02)	0.076

^a^Adjusted for recruitment site, sex, age, income and Western Ontario McMaster Index total score. ^b^ *P* values for Wald test were calculated using robust variance estimates. ^c^Modeled as linear categories (quartiles). *OR* odds ratio

### Determinants of willingness to undergo TKR by race

Table 4 shows the selected determinants of willingness to undergo TKR by patient race. Among WH participants, expectations of TKR (*p* <0.001), TKR utilization process items (*p* = 0.039), and trust in healthcare (*p* = 0.068) were selected for model inclusion. Together, all were statistically significant determinants of willingness to undergo TKR (*p* <0.001). For AA participants, TKR-related knowledge items (*p* = 0.031), expectations of TKR (p = 0.084), religiosity (*p* = 0.045), and trust in physicians (*p* = 0.104) were selected for model inclusion. Together, all were significant determinants of willingness to consider TKR (*p* <0.001).

**Table 4:** Determinants of willingness to undergo total knee replacement (TKR) by race

	White	African-American
	OR (95 % CI)^a^	OR (95 % CI)^a^
Knowledge of TKR		
Understands knee replacement		1.80 (0.97, 3.35)
Length of hospital stay		0.81 (0.58, 1.13)
Extent of pain after recovery		0.73 (0.39, 1.35)
Extent of difficulty walking after recovery		0.66 (0.37, 1.16)
Expectations of TKR^b^		
Second quartile	1.70 (0.86, 3.33)	1.85 (0.76, 4.51)
Third quartile	2.73 (1.32, 5.65)	2.82 (1.30, 6.15)
Highest quartile	5.11 (2.31, 11.30)	2.08 (0.91, 4.79)
TKR process		
Doctor ever discussed surgery	1.96 (1.05, 3.68)	
Referred to surgeon	0.56 (0.32, 0.99)	
Religiosity^b^		
Second quartile		2.52 (0.93, 6.84)
Third quartile		1.21 (0.43, 3.42)
Highest quartile		0.85 (0.32, 2.26)
Trust in physician^b^		
Second quartile		2.14 (0.89, 5.15)
Third quartile		1.01 (0.46, 2.25)
Highest quartile		2.17 (0.90, 5.23)
Trust in healthcare system^b^		
Second quartile	2.69 (1.26, 5.76)	
Third quartile	1.98 (0.91, 4.13)	
Highest quartile	1.58 (0.75, 3.31)	
Likelihood ratio test, *p* value	<0.001	<0.001

Items were selected from a larger set of variables via stepwise regression. ^a^Adjusted for recruitment site, sex, age, income and Western Ontario McMaster Index total score. ^b^Lowest quartile is reference group. *OR* odds ratio

### Mediation of the association between race and willingness to undergo TKR

Multivariable associations between race and willingness to undergo TKR are presented in Table 5. When adjusted for recruitment site, sex, age, income, WOMAC total score, health insurance, and social support, the odds of willingness to undergo TKR was 57 % lower in AAs compared to WHs (OR 0.43, 95 % CI 0.28, 0.67). Addition of the five selected TKR-related knowledge items attenuated this OR towards the null (OR 0.54, 95 % CI 0.34, 0.87). Although the OR was attenuated towards the null for the unadjusted full model (OR 0.51, 95 % CI 0.34, 0.78 vs. OR 0.41, 95 % CI 0.30, 0.57), this was no longer true after adjustment for the covariates (OR 0.45, 95 % CI 0.27, 0.76).

**Table 5:** Mediation of association between race and willingness to undergo total knee replacement (TKR)

	Unadjusted	Adjusted^a^	Adjusted^b^
	OR (95 % CI)	OR (95 % CI)	OR (95 % CI)
Primary predictor, race	0.41 (0.30, 0.57)	0.45 (0.29, 0.70)	0.43 (0.28, 0.67)
Potential mediators			
Knowledge of TKR^c^	0.57 (0.39, 0.82)	0.55 (0.35, 0.88)	0.54 (0.34, 0.87)
Expectations regarding TKR	0.44 (0.32, 0.62)	0.47 (0.30, 0.72)	0.44 (0.28, 0.70)
TKR process^d^	0.41 (0.29, 0.57)	0.42 (0.26, 0.66)	0.39 (0.26, 0.62)
Religiosity	0.41 (0.29, 0.57)	0.43 (0.27, 0.67)	0.42 (0.26, 0.66)
Trust in physician	0.41 (0.30, 0.57)	0.44 (0.28, 0.67)	0.43 (0.27, 0.66)
Trust in healthcare system	0.42 (0.30, 0.58)	0.45 (0.29, 0.69)	0.43 (0.27, 0.67)
Full model^e^	0.51 (0.34, 0.78)	0.45 (0.27, 0.74)	0.45 (0.27, 0.76)

^a^Adjusted for recruitment site, sex, age, income and Western Ontario McMaster Index totalscore. ^b^Additionally adjusted for private/group insurance and social support. ^c^Knowledge items selected for model inclusion: family/friend had hip or knee surgery, understands knee replacement, length of hospital stay, extent of pain after recovery, extent of difficulty walking after recovery. ^d^TKR process items selected for model inclusion: doctor ever discussed surgery, referred to arthritis specialist, referred to surgeon. ^e^Full model includes knowledge of TKR (family or friends who had hip or knee surgery, good understanding of knee replacement, hospital length of stay after TKR, residual pain after recovery, residual difficulty walking after recovery), expectations of TKR (quartiles), TKR process items (ever discussed TKR with a physician, referred to arthritis specialist, referred to surgeon), religiosity (quartiles), trust in physician (quartiles), trust in healthcare system (quartiles). *OR* odds ratio

## Discussion

In this large sample of AA and WH patients with knee OA, we found that determinants of patient preference for TKR differed between AAs and WHs. For AAs, better understanding of the procedure, a shorter hospital course, less post-surgical pain and walking difficulty, favorable expectations of surgery, less religiosity, and trust in physicians significantly influenced patient willingness to undergo TKR. On the other hand, favorable expectations of surgical outcomes, trust in healthcare, discussion with a physician, and not having received surgical referral were significant predictors of willingness among WHs. After controlling for socio-demographic and clinical factors, AAs were still much less willing to consider TKR surgery compared to WHs. Comprehending the process and outcomes of undergoing TKR attenuated this racial difference in patient preference.

Our study has various distinguishing characteristics from previous studies that examined racial differences in OA patients’ treatment preferences. Early studies showed that AAs were less willing to consider joint replacement than WHs based on surveys of male veterans with knee OA [12, 13]. Subsequent studies had similar findings based on surveys of patients recruited from community-based clinics in Texas [20, 37] and North Carolina [21]. In contrast, our study surveyed patients regularly treated at academic facilities, the VA and community-based clinics. Hence, we were able to assemble a cohort of OA patients who differ in regards to gender, socioeconomic status and access to care. Several studies have also explored determinants of treatment preferences for joint replacement [15, 16, 20, 21]. These studies focused on the roles of socio-demographic variables and patient knowledge of joint replacement, but minimally examined the roles of certain variables, including social support, religiosity and/or utilization process items. Finally, while previous studies merely controlled for patient race in their examinations of determinants of treatment preferences [12, 20, 21], our study is the first to identify specific determinants of the willingness of patients with knee OA to undergo TKR, by patient race.

Finding that good understanding of joint replacement and minimal perceptions of pain and difficulties after surgery were determinants of willingness to undergo TKR among AAs is consistent with previous OA studies [12, 16, 20]. Albeit, the North Carolina study [21] found that perceptions of joint replacement outcomes were not significantly associated with willingness to undergo knee or hip replacement surgery. Regardless, better knowledge of the process and outcomes of joint replacement seems to also minimize the racial difference in patient preference for the procedure.

In similar fashion, the knee OA VA study suggested that differences in expectations of hospital course, pain and function after joint replacement fully mediated racial differences in willingness to consider joint replacement [12]. Our study did not support this specific finding of the VA study. Our study did link greater expectations with increased willingness to consider TKR in AAs and WHs with OA separately, however.

We also found that minimal levels of religiosity determined willingness among AAs to undergo TKR. Indeed, many studies have shown that prayer is a common self-care treatment used by AAs [13, 38, 39]. Perceiving prayer as helpful also accounted for differences between AA and WH veterans in their attitudes toward TKR [13]. In contrast, in the Texas study strength in religious belief was unrelated to patient preference for TKR [20]. However, this latter study used a single-item rather than a four-item, ordinal scale to measure the level of religiosity, and multi-item scales tend to outperform single items in terms of predictive validity [40].

Trust in physicians was relevant in AA OA patients’ treatment preferences for TKR. In parallel, trust in healthcare was important in WH OA patients’ preferences. The role of patient trust in providers and the healthcare system in determining preferences has been observed in studies of patients with OA and other diseases [20, 41]. Moreover, mistrust of healthcare organizations and health professionals had been associated with less utilization of health services in other patient populations [42, 43].

The implications of our findings are highly relevant. Decision aids and other educational tools may be designed to educate patients about the benefits and risks of the procedure, and expectations for during and after the procedure. Albeit limited, a few patient-centered educational programs to address such matters have already been developed [44]. Educational strategies and training targeted towards healthcare professionals may also be developed to improve patient trust in physicians and the healthcare system. As a consequence, we may improve OA patients’ preference towards more effective and evidence-based treatments, including TKR. Eventually, we may be able to personalize intervention programs and reduce racial disparities in the utilization of TKR among OA patients.

There are limitations to consider in interpreting the study results. First, the cross-sectional design of this study limits our ability to infer a causal relationship. For instance, we cannot determine whether unwillingness to undergo TKR among WHs was due to prior surgical referral or whether not receiving a surgical referral for TKR was due to unwillingness to have the procedure done. The latter is more likely than the former, as patient preferences for treatments for OA were likely to have been established during the early stages of the disease. Second, we did not determine whether patients were anticipating TKR in the near future. Participants who were in this situation might have been more willing to undergo surgery than patients for whom TKR was a mere hypothetical option. This question was excluded from the survey to minimize patient burden. Third, we included only AAs and WHs, and our findings may not be generalizable to patients from other racial groups. Evaluation of the treatment preferences among other racial and ethnic minorities should be conducted in the future.

## Conclusions

We have identified relatively modifiable factors that determine patient willingness to consider joint replacement, by patient race. Expectations of joint replacement surgical outcomes were associated with preferences for TKR in both races. Adequate understanding of the procedure, minimal perceptions of pain and difficulties after surgery, and higher trust in physicians determined willingness to undergo joint replacement among AAs. Among WHs, trust in the healthcare system and having a surgical discussion with a physician also determined willingness. The findings of this study may lead to the development of highly specific programs that could reduce or possibly eliminate racial and ethnic disparities in the utilization of joint replacement surgery and other clinical outcomes of patients with OA. Hence, we may be able to enhance the quality of care of all patients with this debilitating disease.

## Additional files

Additional file 1:
**Knowledge, attitudes and beliefs of** **African-American** **OA patients by willingness to undergo TKR Surgery.** (DOCX 25 kb) Additional file 2:
**Knowledge, attitudes and beliefs of White OA patients by willingness to undergo TKR Surgery.** (DOCX 25 kb) Additional file 3:
**Logistic regression analyses of willingness and race, stratified by sex.** (DOCX 25 kb) Additional file 4:
**Significant associations (p < 0.20) of willingness to undergo TKR surgery with TKR knowledge and utilization process items.** (DOCX 15 kb)

## Abbreviations

AA: African-American
CI: confidence interval
HSS: Hospital for Special Surgery
MOS-SSS: Medical Outcomes Study-social support scale
OA: osteoarthritis
OR: odds ratio
PHQ-9: Patient Health Questionnaire-9
SF12: Short Form Health Survey
TKR: total knee replacement
VA: Veterans Affairs
WH: white
WOMAC: Western Ontario McMaster Index
