# Supplementary material for: Determinants of patient preferences for total knee replacement: African-Americans and whites
Source: Arthritis Res Ther. 2015 Dec 3;17:348. doi: 10.1186/s13075-015-0864-2 (PMC4669671; doi:10.1186/s13075-015-0864-2)
Supplement: Additional file 1: — Knowledge, attitudes and beliefs of African-American OA patients by willingness to undergo TKR Surgery. (DOCX 25 kb) [file 13075_2015_864_MOESM1_ESM.docx]

Additional file 1. Knowledge, attitudes and beliefs of African-American OA patients by willingness to undergo TKR Surgery

|  | **Not Willing (n=106)** | **Willing**  **(n=176)** | **p-value** |
| --- | --- | --- | --- |
| Religiosity, mean ± SD | 11.13 ± 2.96 | 10.74 ± 2.74 | 0.124 |
| Knowledge of TKR: Familiarity, n (%) Yes | | | |
| Heard about hip or knee surgery | 75 (72.1) | 123 (70.3) | 0.745 |
| Family or friend who had hip or knee surgery | 74 (69.8) | 136 (77.3) | 0.164 |
| Good understanding of knee replacement | 44 (41.5) | 85 (49.4) | 0.199 |
| Knowledge of TKR: Risks & Benefits | | | |
| How often death from knee replacement, n (%) |  |  | 0.429 |
| Never | 20 (19.8) | 36 (22.0) |  |
| Extremely Rare | 63 (61.4) | 105 (64.0) |  |
| Sometimes | 16 (15.8) | 22 (13.4) |  |
| Often | 3 (3.0) | 1 (0.6) |  |
| How long in hospital after knee replacement, n (%) |  |  | 0.151 |
| 1 to 3 days | 18 (17.3) | 42 (24.7) |  |
| 4 to 7 days | 37 (35.6) | 68 (40.0) |  |
| 1 to 2 weeks | 34 (32.7) | 36 (21.2) |  |
| > 2 weeks | 15 (14.4) | 24 (14.1) |  |
| How long to recover from knee replacement, n (%) |  |  | 0.630 |
| < 2 weeks | 0 (0.0) | 2 (1.2) |  |
| 2 weeks to 1 month | 6 (5.9) | 11 (6.5) |  |
| 1 to 2 months | 20 (19.6) | 39 (22.9) |  |
| 2 to 6 months | 32 (31.4) | 50 (29.4) |  |
| 6 to 12 months | 31 (30.4) | 55 (32.4) |  |
| > 12 months | 13 (12.8) | 13 (7.7) |  |
| How much pain after recovery, n (%) |  |  | 0.008 |
| None | 4 (3.9) | 19 (11.4) |  |
| A little | 33 (32.0) | 67 (40.1) |  |
| A moderate amount | 57 (55.3) | 77 (46.1) |  |
| An extreme amount | 9 (8.7) | 4 (2.4) |  |
| How much difficulty walking after recovery, n (%) |  |  | 0.022 |
| None | 4 (3.9) | 23 (13.5) |  |
| A little | 44 (42.3) | 80 (47.1) |  |
| A moderate amount | 50 (48.1) | 58 (34.1) |  |
| An extreme amount | 6 (5.8) | 9 (5.3) |  |
| Expectations regarding TKR, mean ± SD | 45.18 ± 16.75 | 52.28 ± 14.19 | <0.001 |
| TKR Utilization Process, n (%) Yes | | | |
| Doctor ever discuss surgery | 38 (36.2) | 58 (33.1) | 0.603 |
| Referred to arthritis specialist | 37 (35.6) | 70 (40.7) | 0.398 |
| Referred to surgeon | 49 (46.7) | 76 (43.9) | 0.657 |
| Doctor recommended surgery | 26 (24.5) | 41 (23.6) | 0.854 |
| Trust in Physicians, mean ± SD | 38.77 ± 6.69 | 40.08 ± 6.06 | 0.092 |
| Trust in Healthcare System, mean ± SD | 27.06 ± 6.00 | 25.99 ± 5.76 | 0.137 |
