# Supplementary material for: Determinants of patient preferences for total knee replacement: African-Americans and whites
Source: Arthritis Res Ther. 2015 Dec 3;17:348. doi: 10.1186/s13075-015-0864-2 (PMC4669671; doi:10.1186/s13075-015-0864-2)
Supplement: Additional file 2: — Knowledge, attitudes and beliefs of White OA patients by willingness to undergo TKR Surgery. (DOCX 25 kb) [file 13075_2015_864_MOESM2_ESM.docx]

Additional file 2. Knowledge, attitudes and beliefs of White OA patients by willingness to undergo TKR Surgery

|  | **Not Willing (n=102)** | **Willing**  **(n=409)** | **p-value** |
| --- | --- | --- | --- |
| Religiosity, mean ± SD | 9.22 ± 4.28 | 9.09 ± 4.35 | 0.800 |
| Knowledge About TKR: Familiarity, n (%) Yes | | | |
| Heard about hip or knee surgery | 83 (81.4) | 335 (82.7) | 0.750 |
| Family or friend who had hip or knee surgery | 86 (84.3) | 362 (88.5) | 0.249 |
| Good understanding of knee replacement | 62 (60.8) | 280 (70.4) | 0.064 |
| Knowledge About TKR: Risks & Benefits | | | |
| How often death from knee replacement, n (%) |  |  |  |
| Never | 9 (9.3) | 39 (9.8) | 0.911 |
| Extremely Rare | 72 (74.2) | 301 (75.6) |  |
| Sometimes | 16 (16.5) | 57 (14.3) |  |
| Often | 0 (0.0) | 1 (0.25) |  |
| How long in hospital after knee replacement, n (%) |  |  | 0.899 |
| 1 to 3 days | 39 (39.8) | 157 (38.8) |  |
| 4 to 7 days | 42 (42.9) | 182 (44.9) |  |
| 1 to 2 weeks | 11 (11.2) | 48 (11.9) |  |
| > 2 weeks | 6 (6.1) | 18 (4.4) |  |
| How long to recover from knee replacement, n (%) |  |  | 0.794 |
| < 2 weeks | 1 (1.0) | 1 (0.3) |  |
| 2 weeks to 1 month | 3 (3.1) | 19 (4.7) |  |
| 1 to 2 months | 24 (24.7) | 101 (24.8) |  |
| 2 to 6 months | 46 (47.4) | 183 (45.0) |  |
| 6 to 12 months | 19 (19.6) | 91 (22.4) |  |
| > 12 months | 4 (4.1) | 12 (3.0) |  |
| How much pain after recovery, n (%) |  |  | 0.149 |
| None | 13 (13.4) | 68 (17.0) |  |
| A little | 41 (42.3) | 200 (50.1) |  |
| A moderate amount | 41 (42.3) | 128 (32.1) |  |
| An extreme amount | 2 (2.1) | 3 (0.8) |  |
| How much difficulty walking after recovery, n (%) |  |  |  |
| None | 10 (10.0) | 97 (24.1) | <0.001 |
| A little | 54 (54.0) | 226 (56.2) |  |
| A moderate amount | 34 (34.0) | 76 (18.9) |  |
| An extreme amount | 2 (2.0) | 3 (0.8) |  |
| Expectations regarding TKR, mean ± SD | 43.76 ± 17.83 | 53.88 ± 13.76 | <0.001 |
| TKR Utilization Process, n (%) Yes | | | |
| Doctor ever discuss surgery | 27 (26.7) | 155 (37.9) | 0.036 |
| Referred to arthritis specialist | 30 (30.3) | 102 (25.3) | 0.306 |
| Referred to surgeon | 52 (52.0) | 192 (48.4) | 0.516 |
| Doctor recommended surgery | 21 (21.0) | 89 (21.9) | 0.841 |
| Trust in Physicians, mean ± SD | 38.47 ± 6.85 | 39.63 ± 6.89 | 0.127 |
| Trust in Healthcare System, mean ± SD | 25.11 ± 6.21 | 25.04 ± 5.45 | 0.916 |
