# Supplementary material for: Determinants of patient preferences for total knee replacement: African-Americans and whites
Source: Arthritis Res Ther. 2015 Dec 3;17:348. doi: 10.1186/s13075-015-0864-2 (PMC4669671; doi:10.1186/s13075-015-0864-2)
Supplement: Additional file 3: — Logistic regression analyses of willingness and race, stratified by sex. (DOCX 25 kb) [file 13075_2015_864_MOESM3_ESM.docx]

Additional file 3. Logistic regression analyses of willingness and race, stratified by sex

|  |  | **Unadjusted** | **Adjusted** | **Adjusted+ §** |
| --- | --- | --- | --- | --- |
|  | **Race** | **OR (95% CI)** | **OR (95% CI)** | **OR (95% CI)** |
| **Main Analysis (All Subjects) *** | | | | |
|  | African-American | 0.41 (0.30, 0.57) | 0.45 (0.29, 0.70) | 0.43 (0.28, 0.67) |
|  | White |  |  |  |
| **Males †** | | | | |
|  | African-American | 0.29 (0.16, 0.52) | 0.27 (0.13, 0.55) | 0.29 (0.14, 0.61) |
|  | White |  |  |  |
| **Females ‡** | | | | |
|  | African-American | 0.50 (0.34, 0.75) | 0.58 (0.34, 1.00) | 0.55 (0.32, 0.95) |
|  | White |  |  |  |

| *Adjusted model includes recruitment site (VA or non-VA), sex, age, income, and WOMAC total score |
| --- |
| †Adjusted model includes recruitment site (VA or non-VA), age, income, and WOMAC total score |
| ‡Adjusted model includes age, income, and WOMAC total score |
| § Adjusted+ models: further adjusted for social support and private or group insurance (yes or no) |
| Likelihood ratio p-value for interaction of race and sex: p = 0.1164 (unadjusted); p = 0.0394 (adjusted); p = 0.0882 (adjusted+) |
