# Supplementary material for: Determinants of patient preferences for total knee replacement: African-Americans and whites
Source: Arthritis Res Ther. 2015 Dec 3;17:348. doi: 10.1186/s13075-015-0864-2 (PMC4669671; doi:10.1186/s13075-015-0864-2)
Supplement: Additional file 4: — Significant associations (p < 0.20) of willingness to undergo TKR surgery with TKR knowledge and utilization process items. (DOCX 15 kb) [file 13075_2015_864_MOESM4_ESM.docx]

Additional file 4. Significant associations (p<0.20) of willingness to undergo TKR surgery with TKR knowledge and utilization process items.

|  | **All** | **White** | **African-American** |
| --- | --- | --- | --- |
|  | **OR**  **(95% CI)*** | **OR**  **(95% CI)*** | **OR**  **(95% CI)*** |
| Knowledge About TKR† | | | |
| Family or friend had hip/knee surgery | 1.47  (0.93, 2.34) | 1.62  (0.80, 2.38) |  |
| Understands knee replacement | 1.45  (1.00, 2.11) |  | 1.79  (1.00, 3.22) |
| Length of hospital stay |  |  | 0.76  (0.57, 1.03) |
| Extent of pain after recovery | 0.75  (0.53, 1.07) |  | 0.59  (0.32, 1.11) |
| Extent of difficulty walking after  recovery | 0.63  (0.45, 0.88) | 0.64  (0.44, 0.94) | 0.59  (0.33, 1.04) |
| Likelihood Ratio Test p-value | <0.0001 | 0.0004 | 0.0303 |
| TKR Utilization Process† | | | |
| Doctor ever discuss surgery | 1.50  (0.99, 2.29) | 2.07  (1.11, 3.85) |  |
| Referred to arthritis specialist |  |  | 1.44  (0.84, 2.49) |
| Referred to surgeon | 0.72  (0.49, 1.05) | 0.56  (0.32, 0.97) |  |
| Likelihood Ratio Test p-value | 0.1717 | 0.1620 | 0.0739 |

*Adjusted for recruitment site, sex, age, income and WOMAC total score.

† Two separate multivariable models based on stepwise regression
